# Beware of Radiological Diagnosis of Pancreatic Pseudocyst: Lesson Learned From an Interesting Case

**DOI:** 10.7759/cureus.113903

**Published:** 2026-08-03

**Authors:** Nanthesh Kumaran S., Sankar S., Chinni Vikram

**Affiliations:** 1 Surgical Gastroenterology, Sri Ramachandra Institute of Higher Education and Research, Chennai, IND

**Keywords:** cystogastrostomy, differential diagnosis, mucinous cystic neoplasm, pancreatic pseudocyst, pancreatic surgery

## Abstract

Pancreatic pseudocysts are the most common cystic lesions of the pancreas; however, several cystic neoplasms may closely mimic their radiological appearance. Misdiagnosis may result in inappropriate drainage procedures and delayed definitive treatment. A case of pancreatic mucinous cystic neoplasm (MCN) that was repeatedly diagnosed as a pancreatic pseudocyst before definitive surgical management is presented.

A 40-year-old woman with a history of acute pancreatitis presented with recurrent upper abdominal pain and a large pancreatic cystic lesion initially diagnosed as a pancreatic pseudocyst. She underwent endoscopic retrograde cholangiopancreatography (ERCP) with pancreatic duct stenting followed by cystogastrostomy. Despite these interventions, the lesion persisted. Contrast-enhanced CT (CECT) at Sri Ramachandra Institute of Higher Education and Research, Chennai, demonstrated a large cystic lesion in the body and tail of the pancreas with enhancing internal septations, raising suspicion for a cystic neoplasm. The patient subsequently underwent an open distal pancreatectomy with splenectomy. Histopathological examination revealed an MCN characterized by mucin-producing columnar epithelium and ovarian-type stroma, confirming the diagnosis.

This case highlights the diagnostic challenges in differentiating pancreatic pseudocysts from MCNs based on imaging alone. Persistent or recurrent cystic lesions, particularly in middle-aged women and those demonstrating enhancing internal septations, should prompt reconsideration of the diagnosis and further evaluation to facilitate timely surgical management.

## Introduction

Pancreatic cystic lesions encompass a broad spectrum of pathological entities ranging from entirely benign inflammatory collections to premalignant and malignant neoplasms [1]. The most frequently encountered cystic lesion of the pancreas is the pseudocyst, which arises as a consequence of acute or chronic pancreatitis or pancreatic trauma [2]. They occur in approximately 5%-16% of patients after acute pancreatitis and 10%-30% of those with chronic pancreatitis. Histologically, it is characterised by an encapsulated collection of pancreatic secretions surrounded by a fibrous wall lacking an epithelial lining and is commonly associated with disruption of the main pancreatic duct [3].

The differential diagnosis of pancreatic cystic lesions includes walled-off necrosis (WON), serous cystadenoma (SCA), mucinous cystic neoplasm (MCN), intraductal papillary mucinous neoplasm (IPMN), and solid pseudopapillary neoplasm (SPN) [4]. Among these, MCN is of particular clinical importance because of its malignant potential. Women account for approximately 98% of MCN cases, with most patients presenting in the fifth decade of life. Histologically, MCN is characterised by mucin-producing epithelium supported by ovarian-type stroma [5].

The radiological features of MCN and pseudocyst overlap considerably, and both may appear as unilocular or multilocular cystic masses in the body or tail of the pancreas. This overlap can result in misdiagnosis, with inappropriate drainage procedures performed in place of oncological resection. The distinction is of critical importance: While pseudocysts may be managed conservatively or by internal drainage, MCNs require surgical resection due to their premalignant nature [6].

This case describes a pancreatic MCN that was initially interpreted as a pancreatic pseudocyst and managed with multiple drainage procedures before definitive diagnosis was established following surgical resection and histopathological examination.

## Case presentation

A 40-year-old woman presented to the outpatient department with upper abdominal pain and fever with chills of one week duration. On physical examination, a mass was palpable in the epigastrium. Blood investigations were within normal limits.

She had a history of abdominal pain, vomiting, and loss of weight and appetite, and was diagnosed with acute pancreatitis at an outside hospital. CT of the abdomen was suggestive of a pancreatic pseudocyst measuring 9 × 8.5 cm. She subsequently underwent endoscopic retrograde cholangiopancreatography (ERCP) with pancreatic duct stenting. Repeat ultrasound examination of the abdomen demonstrated a 9 × 9 cm anechoic lesion in the epigastric region with internal echoes, following which the stent was removed. Follow-up CT imaging again demonstrated a cystic lesion involving the body and tail of the pancreas, which was reported as a pancreatic pseudocyst. She subsequently underwent cystogastrostomy for management of the presumed pancreatic pseudocyst. Following surgery, she developed low back pain and was later lost to follow-up.

**Figure 1:**
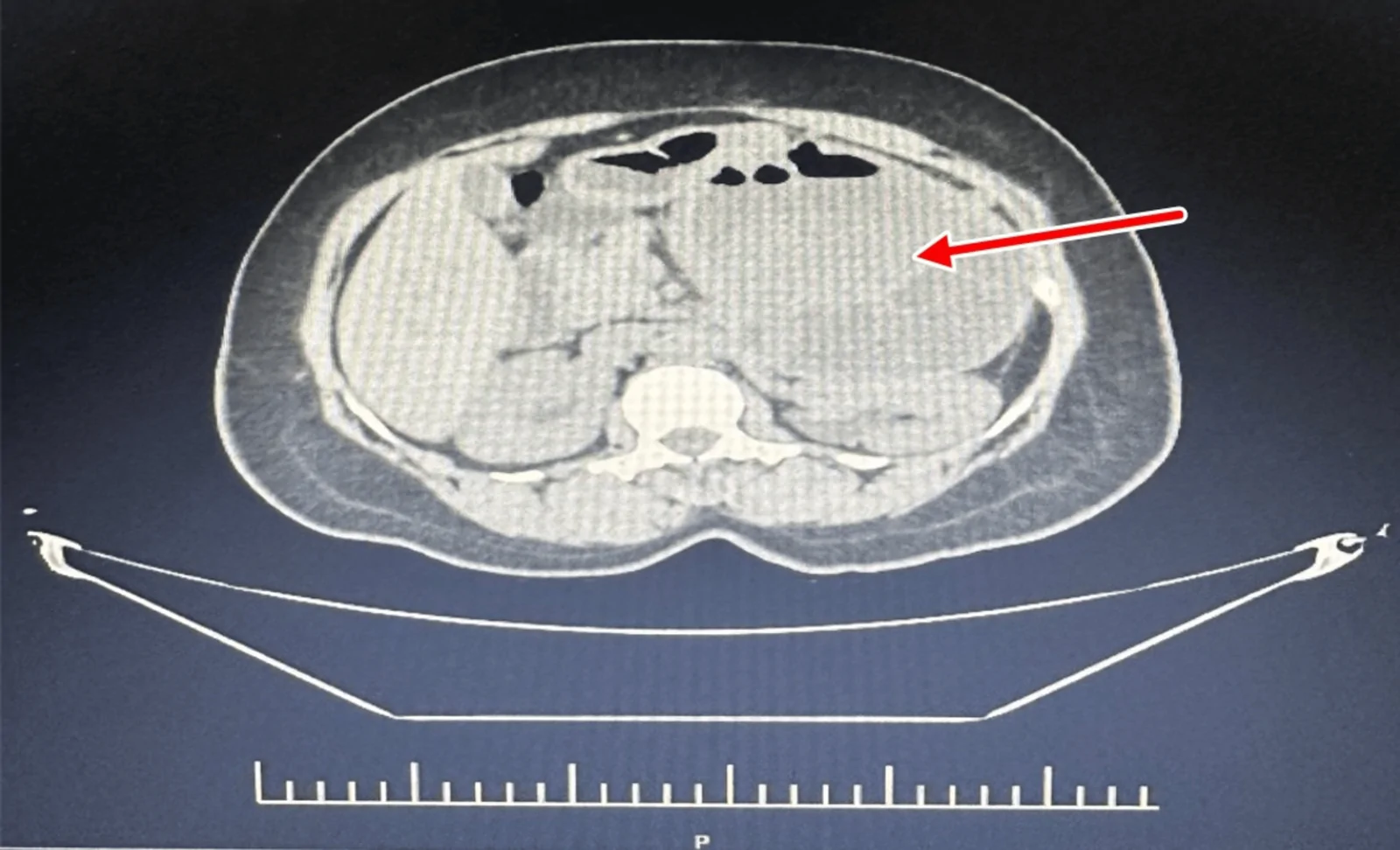
Plain CT image of the abdomen showing a large cystic lesion involving the body and tail of the pancreas (red arrow indicates the cystic pancreatic lesion)

**Figure 2:**
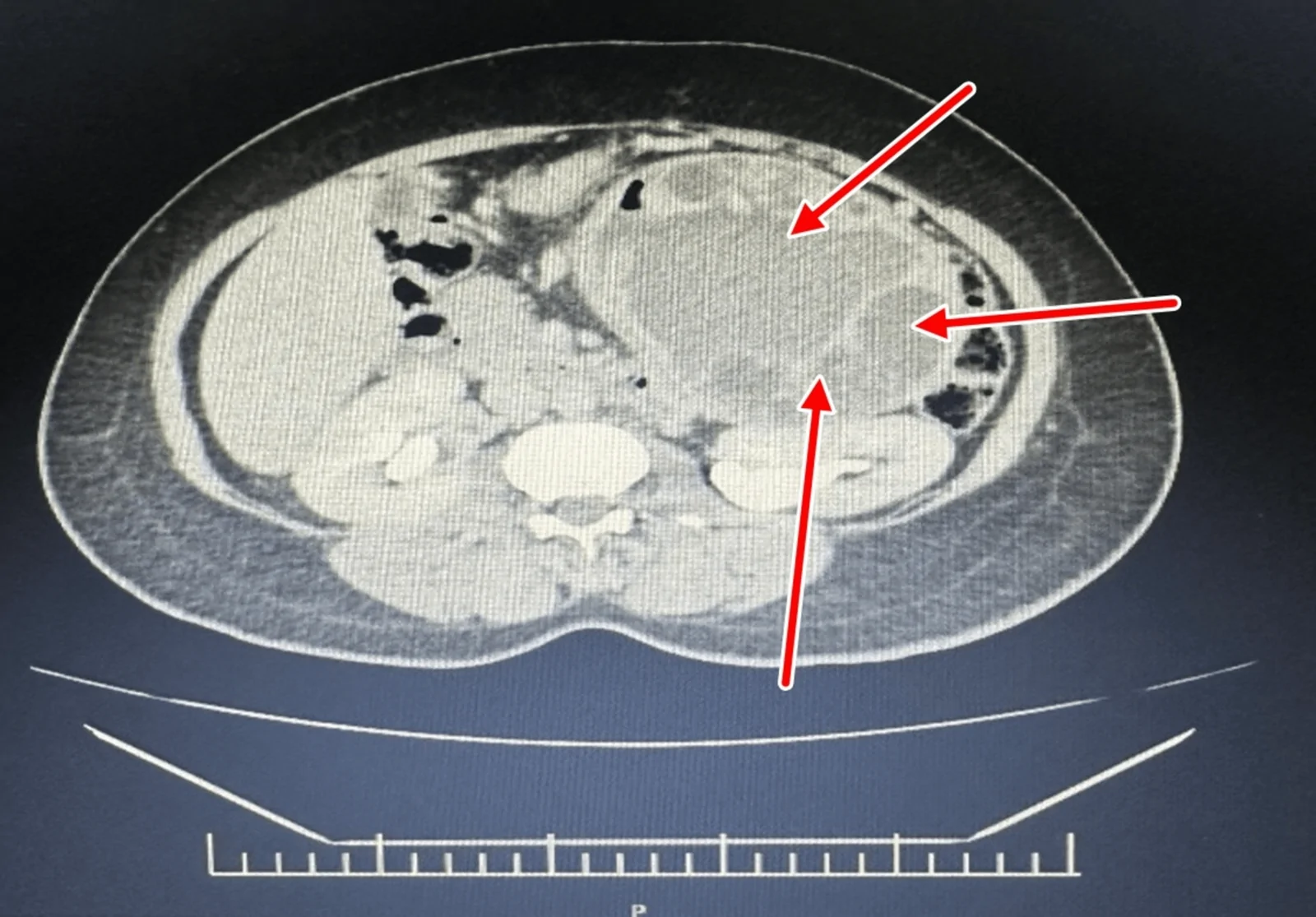
Arterial-phase CECT image of the abdomen demonstrating multiple enhancing internal septa within the cystic lesion (red arrows indicate the enhancing internal septa) CECT: Contrast-enhanced CT

Upon evaluation at the present institution, contrast-enhanced CT (CECT) of the abdomen was performed, and the initial radiological impression was again suggestive of a pancreatic pseudocyst measuring 10.4 × 10.8 × 10.3 cm. The plain phase demonstrated a large predominantly cystic lesion (Figure 1). However, the arterial phase revealed multiple enhancing internal septations within the lesion (Figure 2), raising suspicion for a cystic neoplasm rather than a pancreatic pseudocyst.

**Figure 3:**
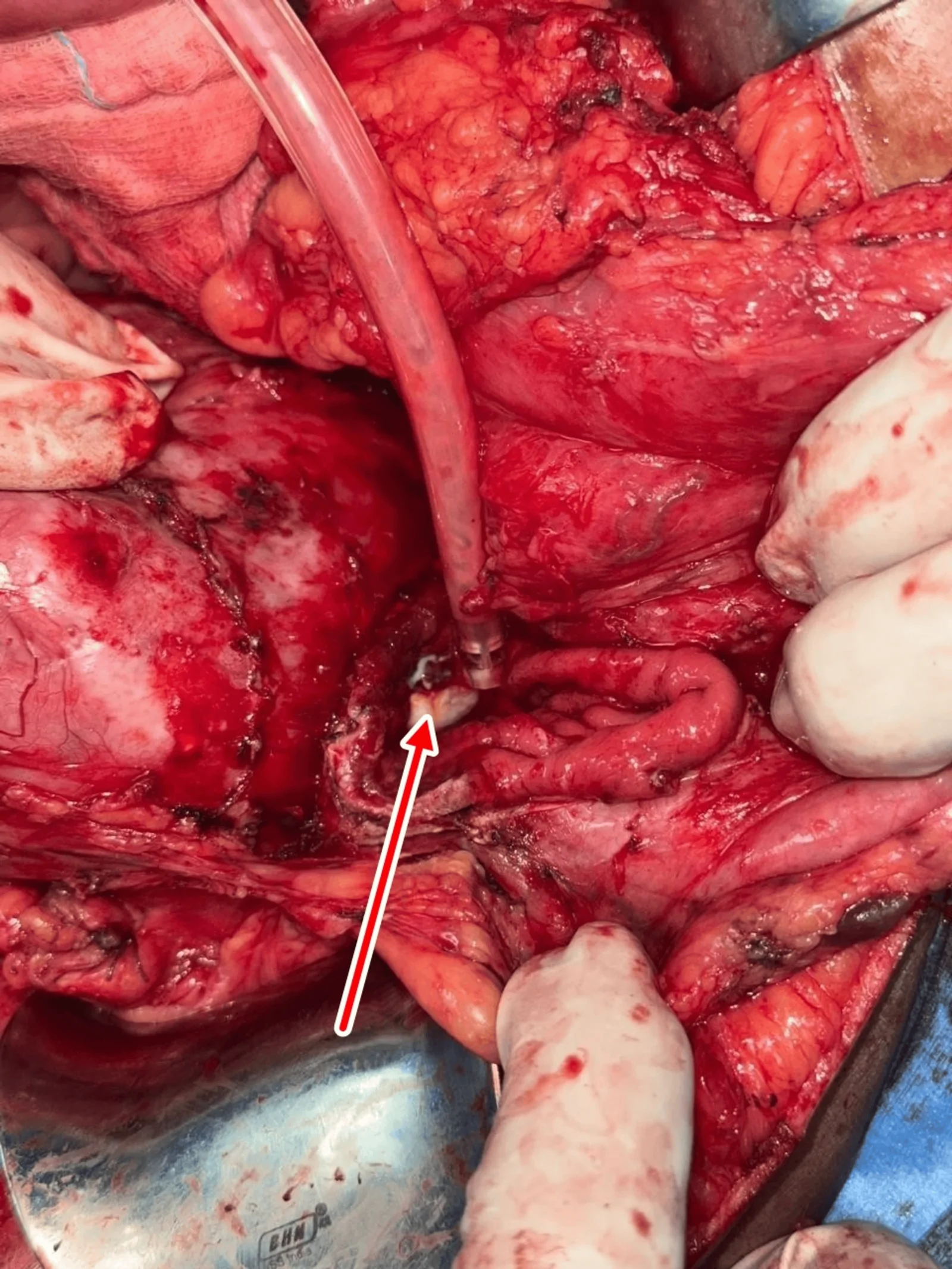
Intraoperative photograph showing a large mass involving the body and tail of the pancreas with mucin extruding from the lesion (red arrow indicates the extruding mucin)

In view of the size of the lesion and the enhancing septated appearance on the arterial phase, the patient was taken for open surgery. She underwent open pancreaticosplenectomy. Intraoperatively, a large mass was found involving the body and tail of the pancreas, with mucin extruding from the mass (Figure 3).

The resected specimen consisted of the distal pancreas and spleen, weighing 1052.5 g and measuring 15.5 x 12.5 x 6.5 cm overall. A single large globular lesion was identified in the distal pancreas, measuring 12.5 x 10.5 x 6.5 cm, with a smooth grey-brown external surface and a focal irregular area measuring 4 x 2.5 x 0.6 cm. On sectioning, thick yellowish-green mucus extruded, and multiple cysts were identified on the cut surface. The largest cystic space measured 11.5 x 9 x 8.5 cm, with a wall thickness ranging from 1.0 to 1.8 cm and a grey-white firm internal surface devoid of solid or papillary areas. The remaining cysts ranged from 0.2 to 3 cm in size, with wall thickness of 0.2 to 0.3 cm, smooth internal surfaces, and no solid or papillary areas. The spleen measured 13 x 10 x 4 cm and was unremarkable, attached to the lesion by fibrofatty tissue measuring 4.5 x 3 x 1 cm.

Microscopic examination revealed multiple cysts lined by flattened cuboidal to mucinous columnar epithelium, with the lining thrown into folds in some areas. The lining cells exhibited moderate eosinophilic cytoplasm and basally located bland nuclei, without significant atypia or mitotic activity. Some cysts were filled with mucin and others with eosinophilic material. The cysts were surrounded by ovarian-type stroma. The adjacent pancreatic parenchyma showed dense fibrosis with architectural distortion, loss of acini, scattered pancreatic islets, and dense lymphoplasmacytic infiltration with focal granulation tissue formation. The adjacent fibroadipose tissue showed scattered lymphocytes and occasional congested blood vessels. Sections from the focal irregular area on the external surface showed gastric mucosa, consistent with a cuff of gastric wall adherent from the earlier cystogastrostomy.

These histopathological features, particularly the mucin-producing columnar epithelium and the characteristic surrounding ovarian-type stroma, confirmed the diagnosis of MCN of the pancreas. The patient had an uneventful postoperative recovery, tolerated oral diet well, had satisfactory wound healing, and remained asymptomatic at the three-month outpatient follow-up.

**Table 1:** Timeline of the patient's clinical course ERCP: Endoscopic retrograde cholangiopancreatography; CECT: Contrast-enhanced CT; MCN: Mucinous cystic neoplasm

Timeline	Clinical Events
Initial Presentation (Outside Hospital)	Presented with abdominal pain, vomiting, weight loss, and loss of appetite. Diagnosed with acute pancreatitis; CT demonstrated a 9 × 8.5 cm pancreatic cystic lesion, reported as a pancreatic pseudocyst.
Initial Management	ERCP with pancreatic duct stenting performed.
Follow-Up Evaluation	Ultrasound demonstrated a persistent 9 × 9 cm cystic lesion with internal echoes. Repeat CT again suggested a pancreatic pseudocyst, and the pancreatic stent was removed.
Further Management	Cystogastrostomy performed for the presumed pancreatic pseudocyst.
Subsequent Course	Developed low back pain and was lost to follow-up.
Subsequent Evaluation	Presented with upper abdominal pain and fever with chills. CECT demonstrated a 10.4 × 10.8 × 10.3 cm predominantly cystic lesion with enhancing internal septations, raising suspicion for a cystic neoplasm.
Definitive Treatment	Open pancreaticosplenectomy performed. Intraoperatively, mucin extrusion from the lesion was noted.
Final Diagnosis	Histopathological examination confirmed pancreatic MCN.
Outcome	Uneventful postoperative recovery with good oral intake, satisfactory wound healing, and an uncomplicated outpatient follow-up.

## Discussion

This case illustrates the diagnostic challenge of relying solely on radiological assessment for pancreatic cystic lesions. The patient underwent ERCP with pancreatic duct stenting followed by cystogastrostomy under the working diagnosis of a pancreatic pseudocyst. Despite these interventions, the cystic lesion persisted on interval imaging, and the definitive diagnosis of MCN was established only after surgical resection. The diagnosis was confirmed by intraoperative extrusion of mucin and characteristic histopathological features.

MCNs of the pancreas are uncommon tumors, constituting approximately 10%-45% of resected pancreatic cystic neoplasms [7]. They occur almost exclusively in women, with a peak incidence in the fourth and fifth decades of life, and are located predominantly in the body and tail of the pancreas [7]. These demographic and anatomical features were clearly present in the index case; in retrospect, they should have prompted heightened suspicion for a neoplastic rather than an inflammatory etiology from the outset. Fernandez-del Castillo and Warshaw emphasized that the demographic profile of the patient is one of the most important clinical clues in distinguishing MCN from pseudocyst, and that middle-aged women with cystic lesions in the body or tail of the pancreas warrant careful further evaluation before a diagnosis of pseudocyst is accepted [8].

The radiological distinction between MCN and pseudocyst has been studied extensively. Sahani et al. demonstrated that imaging features, such as thick or enhancing walls, enhancing internal septa, mural nodules, and the absence of a clear precipitating cause, should raise suspicion for a cystic neoplasm [9]. In the present case, the arterial phase of the CECT obtained at the present institution clearly demonstrated multiple enhancing septa, which is a well-recognized imaging feature of MCN and is not a feature of a benign pancreatic pseudocyst. Kim et al., in a review of imaging characteristics of cystic pancreatic lesions, similarly identified enhancing septations as a key feature distinguishing mucinous neoplasms from inflammatory pseudocysts [10]. The failure to accord diagnostic significance to these enhancing septa at both the outside institution and on initial review at the present institution reflects the challenge of pattern recognition in this setting.

The persistence of the lesion despite cystogastrostomy is itself diagnostically informative. Pancreatic pseudocysts are generally expected to resolve or remain stable following adequate internal drainage. Persistence of a cystic lesion after drainage should prompt reconsideration of the diagnosis and further evaluation to exclude an underlying cystic neoplasm. Garcea et al. reported that misdiagnosed cystic neoplasms that underwent drainage procedures rather than resection showed disease progression, and recommended that any cystic lesion that fails to resolve or enlarges after drainage be reassessed with more detailed radiological and endoscopic evaluation [11]. The recurrence of the cystic lesion in the present patient following cystogastrostomy, as evidenced by the persistence of the mass at the present institution, is consistent with the natural history of an unresected MCN.

The role of endoscopic ultrasound-guided fine-needle aspiration (EUS-FNA) and cyst fluid analysis in the preoperative characterisation of pancreatic cystic lesions is well established. Brugge et al., in a multicentre trial, demonstrated that intracystic carcinoembryonic antigen (CEA) levels exceeding 192 ng/mL are highly specific for a mucinous cystic lesion, with a sensitivity of 73% and specificity of 84% [12]. The presence of mucin on cytological examination further supports the diagnosis of MCN or IPMN. EUS also allows detailed assessment of wall characteristics, septa, and mural nodules with a resolution superior to cross-sectional imaging. Had EUS-FNA with cyst fluid analysis been performed at the time of initial evaluation, it is probable that the mucinous nature of the lesion would have been identified, thereby directing the patient towards surgical resection rather than drainage.

A limitation of this case is the absence of preoperative endoscopic EUS-FNA with cyst fluid analysis. Nevertheless, the CECT findings were sufficient to prompt surgical resection, although cyst fluid CEA estimation and cytological evaluation might have provided complementary diagnostic information during the preoperative assessment.

The histopathological findings in this case are characteristic of MCN. The presence of mucin-secreting columnar epithelium with bland nuclei and the surrounding ovarian-type stroma are the defining features of this entity, as outlined in the WHO Classification of Digestive Tumours [13]. Ovarian-type stroma is considered pathognomonic for MCN and is not seen in pseudocysts or other pancreatic cystic lesions. The adjacent pancreatic parenchymal changes of dense fibrosis, architectural distortion, loss of acini, and lymphoplasmacytic infiltration reflect chronic obstructive pancreatitis resulting from the mass effect of the neoplasm. The focal irregular area on the external surface of the specimen, showing gastric mucosa on microscopy, represents a cuff of gastric wall adherent from the prior cystogastrostomy, providing direct histological evidence of the previous drainage procedure. Fukushima and Zamboni reviewed the surgical pathology of MCN and confirmed that these histological features, particularly the ovarian stroma and the absence of communication with the pancreatic ductal system, are sufficient to establish the diagnosis without the need for molecular or immunohistochemical confirmation in most cases [14].

The differences in key clinical and radiological features between pancreatic pseudocyst and MCN are summarized in Table 2. The co-existence of the clinical context with imaging features such as enhancing septa should guide the clinician toward further evaluation and away from an unqualified diagnosis of pseudocyst. The Fukuoka guidelines and the European evidence-based guidelines on pancreatic cystic neoplasms both stipulate that worrisome features including enhancing septa, thick walls, and lesion size exceeding 3 cm mandate further evaluation with EUS before a management decision is made [15,16].

**Table 2:** Distinguishing features between pancreatic pseudocyst and MCN CEA: Carcinoembryonic antigen; MCN: Mucinous cystic neoplasm Figure adapted from [3,18,19]

Feature	Pseudocyst	MCN
Precipitating history	Pancreatitis or trauma	Usually absent
Demographics	Any age, any sex	Middle-aged women
Location	Anywhere in pancreas	Body and tail
Wall characteristics	Thin, non-enhancing	Thick, may enhance
Internal septa	Absent or non-enhancing	Enhancing septa present
Solid components	Absent	May be present
Ductal communication	May communicate	Absent
Intracystic CEA	Low (<5 ng/mL)	High (>192 ng/mL)
Intracystic amylase	Usually elevated	Usually low
Epithelial lining	Absent	Mucinous columnar epithelium
Stroma	Fibrous/granulation tissue	Ovarian-type stroma
Response to drainage	Resolution expected	Persistence or enlargement
Malignant potential	None	Present

Open pancreaticosplenectomy, as performed in this case, is the standard surgical procedure for MCN located in the body and tail of the pancreas. Hui et al. reported five-year survival rates approaching 100% for non-invasive MCN following complete surgical resection, underscoring the excellent prognosis when the lesion is resected before malignant transformation [17]. In the present case, the absence of solid or papillary projections, significant nuclear atypia, or mitotic activity is consistent with a non-invasive MCN, and the patient remained asymptomatic, with no evidence of recurrence, at the three-month outpatient follow-up, supporting a favorable prognosis following complete resection.

## Conclusions

MCN should be considered in the differential diagnosis of pancreatic cystic lesions, particularly in middle-aged women with lesions involving the body and tail of the pancreas. Persistence or enlargement of a presumed pseudocyst despite drainage procedures, along with radiological features, such as enhancing internal septa, should prompt reconsideration of the diagnosis and further evaluation. Early recognition and appropriate surgical management are essential to avoid delayed treatment of this premalignant lesion.
